# An Assist-as-Needed Control Strategy Based on a Subjective Intention Decline Model

**DOI:** 10.3390/bioengineering11111113

**Published:** 2024-11-04

**Authors:** Hao Yan, Fangcao Zhang, Xingao Li, Chenchen Zhang, Yunjia Zhang, Yongfei Feng

**Affiliations:** 1 School of Mechanical and Equipment Engineering, Hebei University of Engineering, Handan 056038, China; yanhao@hebeu.edu.cn (H.Y.); wy2002123@126.com (X.L.); zhang2415198975@163.com (C.Z.); 18631579511@163.com (Y.Z.); 2 Orthopedic Medical Robot Hebei Engineering Research Center, Handan 056038, China; 3 School of Mathematics and Physics, Hebei University of Engineering, Handan 056038, China; zhangfangcao@hebeu.edu.cn; 4 Faculty of Mechanical Engineering and Mechanics, Ningbo University, Ningbo 315000, China

**Keywords:** subjective intention attenuation model, quantum particle swarm optimization algorithm, assist-as-needed control strategy

## Abstract

In the rehabilitation training process for stroke patients, the level of excitement in the patient’s physiological state has a positive impact on the efficacy of the training. In order to improve patients’ initiative during training and prevent dependence on assistive systems, this study proposes an assist-as-needed control strategy based on a subjective intention decline model. The strategy primarily consists of two modules: a subjective intention decline control module and a limb movement assessment module. The subjective intention decline module collects surface electromyography (sEMG) data during patient training and optimizes support vector machine (SVM) using quantum particle swarm optimization (QPSO) algorithms to establish a subjective intention decline model. The limb movement assessment module collects information such as interaction force and position error during training and proposes a method for evaluating the motion state of the affected limb. This model combines traditional impedance control with a method for assessing limb movement and subjective status, automatically adjusting the level of assistive force on the affected limb in real time to enhance its active participation in tasks. Finally, we performed two verification experiments to assess the patient’s initiative in participating in the training. The experimental results show that the proposed method effectively reduced the average assist force by 65.66% for the traditional impedance control training system and effectively the average assist force by 35.2% for the control training system using only the assist force module based on force position information. At the same time, the accuracy of the subjective intention attenuation module established in the experiment to identify the fatigue level of the subjects reached 93.41%. Therefore, the proposed method effectively improves the initiative of trainers and also prevents patients from relying on the assist-as-needed control training system.

## 1. Introduction

At present, the application of rehabilitation robots to assist patients in restoring their motor ability has become the main effective way of rehabilitation training, and the application of rehabilitation robots has also made up for the shortage of rehabilitation physicians. Patients’ subjective participation is very important to the clinical effect of upper limb rehabilitation [1,2]. Therefore, the robot must have the ability to understand the human motion intention. At present, most rehabilitation robots use a variety of control strategies to integrate the patient’s behavior intention into the robot control loop in order to improve the patient’s active participation in the training process [3,4,5,6]. Current control strategies mainly include admittance control, impedance control, adaptive assist force control, etc. [7,8]. Guo Bingjing proposed an adaptive assist control strategy to quantitatively evaluate the interactive control in the task-oriented space. This method can estimate patients’ exercise needs effectively, achieve progressive rehabilitation training, and motivate patients to participate actively and voluntarily in upper limb rehabilitation treatment [9]. Li Huijun proposed an upper limb mirroring control strategy based on an adaptive assist-as-needed strategy [10], which automatically adjusts the assist force of the affected limb in real time to maximize the reduction in the active torque of the affected limb. Guo Yida proposed an assist-as-needed control strategy based on position/force evaluation to stimulate the active participation of subjects in the training process [11]. The outer loop of the control system uses a fuzzy adaptive impedance controller, and the inner loop uses a combination of radial basis functions to control the assist force required by the exoskeleton. Yan Hao proposed a support vector machine fusion QPSO method to assess the degree of patient participation in training tasks [12]. However, this predictive model has certain limitations as it only uses force and position data. The above assist-as-needed force control strategy provides as little assist force as possible according to the patient’s real-time state needs to help the patient complete the rehabilitation training task. Thus, during the rehabilitation training process, the system provides an assist force to reach the minimum value, and the patient actively provides the muscle strength to reach the maximum value [13,14], thereby enhancing the patient’s initiative and improving the rehabilitation training effect.

Studies have shown that the initiative of stroke patients’ subjective intention to participate in training has a great impact on the rehabilitation effect [15,16]. At present, the parameter design and adjustment of the on-demand assistive force control strategy depend on the expert knowledge and previous experience of the therapist, and there is a lack of quantitative control strategy design methods [17]. At the same time, patients who participate in on-demand assistive training many times will have a dependency on the robot’s assistive force due to their own inertia, so as to “deceive” the robot into generating greater assistive force or generating inappropriate assistance to reduce the patient’s active participation and even miss the opportunity to induce changes in neural plasticity [18]. Therefore, integrating patients’ subjective participation into the control strategy is a necessary condition for the robot to provide optimal assistance [19,20,21].

Therefore, this paper proposes an on-demand assist control strategy based on the subjective intention attenuation model, which adds judgment on the subjective participation level of patients based on previous research on adjusting auxiliary force according to force position data. In this approach, physiological signals are collected from patients during the training process to extract a subjective intention decline control model that evaluate the level of active participation in training. Based on the active participation status, we adjust the auxiliary force required to compensate for the movement of the affected limb in real time. Two comparative experiments were designed to compare the performance differences between the traditional impedance control method, the control training system using only the limb movement assessment module, and the control training system using the proposed method. In the second experiment, the subjects simulated the phenomenon of dependence on the assist force. The subjects were trained using a control training system based on the limb movement assessment module, as well as the control training system proposed in this paper. By analyzing the assist force of the two methods and the task achievement rate after training, the effectiveness of the method in this paper to detect the subjective participation of patients and the effect of reducing the dependence on the assist force were verified.

## 2. On-Demand Assist Control Strategy of Subjective Intention in the Loop

**Figure 1:**
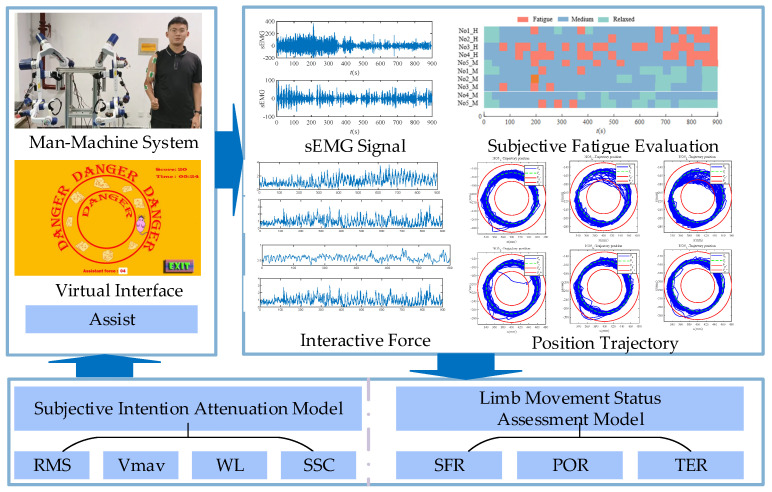
On-demand assist control strategy of subjective intention attenuation model.

In this paper, an assist-as-needed control strategy based on a subjective intention attenuation model is proposed. As shown in Figure 1, the on-demand assist control strategy adopts admittance control. In the control strategy, the robot adjusts the auxiliary force in real time based on the force evaluation parameters and subjective intention attenuation model of the patient’s training state. Subjects participate in rehabilitation training through man–machine systems and virtual interfaces, collecting interaction forces, position trajectories, sEMG signals, and subjective fatigue evaluation data of the subjects during the training process. Based on previous research results, a model for limb movement status assessment is designed. The subjective intention attenuation model is trained based on the subjective fatigue evaluation data of the subjects.

### 2.1. Subjective Intention Attenuation Model

In order to evaluate the degree of patients’ subjective participation intention in training, the implicit mathematical relationship between the sEMG signal and the patient’s subjective intention is established by a support vector machine algorithm, so as to obtain the attenuation state coefficient α of the patient’s subjective intention. The sEMG signal during the training process reflects the patient’s subjective intention stimulation. Because of the difference between individual and subjective acceptance ability, the level of stimulation cannot be used as the basis for providing auxiliary force, so the rate of stimulation decline is selected as the attenuation coefficient of subjective intention. The patient’s subjective intention attenuation model is established, in which the attenuation coefficient α is mainly determined by the following four characteristics:

The average degree of muscle activation, that is, the sEMG signal amplitude root mean square (*RMS*), which is determined as follows:

(1) $$RMS=\sqrt{\frac{1}{T_{n}}\sum_{i=1}^{T_{n}}sEMG_{i}^{2}}$$

Here, $T_{n}$ is the number of data points of the surface sEMG signal in the subjective intention observation window, and $sEMG_{i}$ is the data point of the sEMG signal in the observation window. Patients need stronger EMG signals to complete training tasks when they are in a state of fatigue [22], and *RMS* can reflect this feature well. Therefore, the higher the *RMS* degree of muscles is, the more excited the muscles are. The lower the *RMS* degree is, the more relaxed the muscles are.

Muscle waking speed, that is, the mean absolute value (*MAV*), expressed as follows:

(2) $$MAV=\frac{\sum_{i=1}^{T_{n}}T_{i}s_{i}-T_{n}\bar{T}\cdot \bar{s}}{\sum_{i=1}^{T_{n}}{\left(T_{i}\right)}^{2}-n{\left(\bar{T}\right)}^{2}}$$

where $T_{i}$ is discrete-time data, and $s_{i}$ is the data point of the sEMG signal in the observation window. When the MAV index value is greater than zero, the patient’s subjective intention is in the recovery stage. When the index value is less than zero, the patient’s subjective intention is declining. When the patient works harder to complete the current task, the higher the level of muscle stimulation, the easier the patient is to experience fatigue, and the smaller the MAV; the range of MAV values is limited to (0, 1) by normalization.

sEMG signal complexity, that is, the Weisfeiler–Lehman (*WL*) value, defined as follows:

(3) $$WL=\sum_{i=1}^{T_{n}}\left|sEMG_{i+1}-sEMG_{i}\right|$$

In the subjective intention observation window, by adding the waveform length of the sEMG signal, the complexity of the sEMG signal is expressed. The more complex the sEMG signal is, the more muscles the patient has to mobilize to participate in the training. The corresponding waveform length will also increase, and the patient is more likely to experience fatigue. The *WL* index will also increase accordingly; thus, the *WL* value range is limited to (0, 1) by normalization.

Electromyographic signal volatility, that is, the sparse subspace clustering (*SSC*), which is defined as follows:

(4) $$SSC=\frac{1}{T_{n}}\sum_{i=1}^{T_{n}-1}f\left(i\right),\mathrm{Among}\;\mathrm{them}\;f\left(i\right)=\left\{\begin{array}{cc} 1 & \begin{array}{c} s_{i}>s_{i-1},s_{i}>s_{i+1}\;OR\;s_{i}<s_{i-1},s_{i}<s_{i+1}\\ meanwhile\left|s_{i}-s_{i+1}\right|>s_{th}\;OR\;\left|s_{i}-s_{i-1}\right|>s_{th} \end{array}\\ 0 & else \end{array}\right.$$

The data in $f\left(i\right)$ are discrete data, where $s_{i-1}$, $s_{i}$, and $s_{i+1}$ are the *i* − 1, *i*, and *i* + 1 data in the signal data sequence, respectively. $s_{th}$ is the set noise threshold, and SSC is the number of times the sign of the amplitude change rate changes. When the patient is excited, and the muscle is tense, the muscle signal fluctuates more and the SSC value is larger, which also means that the patient’s subjective intention attenuates faster. The range of SSC values is limited to (0, 1) by normalization.

A mathematical model based on support vector classifiers and regression machines was established according to the characteristics of the small sample and non-linear data derived from a small number of patients’ training and questionnaire data. The support vector machine is used to determine the classification hyperplane ωx+b=0 between two different fatigue state evaluation feature data, and then the subjective intention attenuation coefficient function $\alpha_{i}\left(x\right)=\omega^{Τ}\cdot \Phi \left(x_{i}\right)+b_{i}$ is obtained, where $\Phi \left(x\right)$ is the kernel function, which is used to transform the sample space dimension. Let the sample dataset ${\left\{\left(x_{i},\alpha_{i}\right)\right\}}_{i=1}^{l}\in {\left(R^{n}\times \alpha \right)}^{l}$, $x_{i}\in R^{n}$ be the input value of the characteristic quantity of the i training data, $\alpha_{i}\in \alpha =R$ be the output value of the difficulty evaluation of the i training task, and i=1,2,…,l, *l* be the total number of samples, and introduce the transformation function $\Phi \left(x\right)$ as follows:

(5) $$\Phi :\begin{array}{c} R^{n}\to H\\ x\to X=\Phi \left(x\right) \end{array}$$

Then, the training sample is ${\left\{\left(\Phi \left(x_{i}\right),\alpha_{i}\right)\right\}}_{i=1}^{l}\in {\left(H\times \alpha \right)}^{l}$.

Let the decision function, that is,

(6) $$\alpha_{i}\left(x\right)=\omega^{Τ}\cdot \Phi \left(x_{i}\right)+b_{i},i=1,2,\ldots ,l$$

In this formula, ω is the weight vector and *b* is the adjust parameters.

According to the principle of structural risk minimization, the optimization problem is transformed into the problem of finding the minimum value [23]. The radial basis function is selected as the kernel function in the support vector regression (SVR) model. Combined with Lagrange and Karush–Kuhn–Tucker conditions, the dual equation of the optimization function can be derived. Therefore, the estimation function of SVR can be used to predict the degree of active participation of patients using Formula (6).

(7) $$f\left(x\right)=\sum_{x_{i}\in SV}\left(\alpha_{i}-\alpha_{i}^{*}\right)\left(\Phi (x_{i})\cdot \Phi (x)\right)+b$$

The selection of two key parameters *C* and *σ* in support vector machine affects the generalization ability and accuracy of learning. In order to eliminate the local optimal problem in parameter selection, the QPSO algorithm is introduced to optimize the parameter selection of the support vector machine, forming the QPSO-SVM hybrid algorithm [24]. The feasible solution updating process of the QPSO algorithm is as follows:

(8) $$\left\{\begin{array}{l} m_{best}=\frac{1}{M}\sum_{i=1}^{M}P_{i}\\ P_{C_{ij}}=\phi P_{ij}+\left(1-\phi \right)P_{gj}\\ x_{ij}\left(t+1\right)=P_{C_{ij}}\pm \alpha \left|m_{bestj}-x_{ij}\left(t\right)\right|\ln\left(\frac{1}{u}\right) \end{array}\right.$$

The particle velocity update formula is as follows:

(9) $$v_{ij}\left(t+1\right)=\omega \cdot v_{ij}\left(t\right)+c_{1}r_{1j}\left[P_{ij}\left(t\right)-x_{ij}\left(t\right)\right]+c_{2}r_{2j}\left[P_{gj}\left(t\right)-x_{gj}\left(t\right)\right]$$

In this formula, *P_ij_* and *P_gj_* are the optimal position of the *i*-th particle and the *g*-th particle swarm on the *j*-th dimension, *m*_best_ is the center point of the current best position on all individuals and dimensions, *M* is the size of the particle swarm, *P_i_* is the current optimal position of the *i*-th particle, *P_cij_* is the random position between *P_ij_* and *P_gj_*, *α is* control coefficient, and *T*_max_ is the maximum number of iterations.

Based on the above theory, the model parameters *M* and *T_max_* of QPSO are initialized, and then the two key parameters *C* and *σ* of SVR are optimized by calculating and updating the optimal position and velocity of individual and group particles. The optimization goal is to achieve the minimum value of fitness(σ,γ). The sample mean square error (MSE) is selected as the particle swarm fitness function, that is,

(10) $$fitness\left(\sigma ,\gamma \right)=\frac{1}{M}\sum_{i=1}^{M}{\left(\alpha_{i}-{\hat{\alpha }}_{i}\right)}^{2}$$

In the above formula, *α_i_* is the actual value, and ${\hat{\alpha }}_{i}$ is the predicated value.

### 2.2. Limb Movement Status Assessment Module

The limb movement state assessment module uses the fluctuations in human–machine interaction force and position information during training tasks to evaluate the movement state of the affected limb. Firstly, multiple specific indicators are extracted from the training information, and then a force position mixed evaluation index *β* is designed, which is mainly determined by the following three parameters:

The fluctuation rate of limb status, that is,

(11) $$SFR=\frac{1}{T}\int_{0}^{T}\sqrt{{\left(\frac{d^{2}F_{m}^{x}}{dt^{2}}\right)}^{2}+{\left(\frac{d^{2}F_{m}^{y}}{dt^{2}}\right)}^{2}+{\left(\frac{d^{2}F_{m}^{z}}{dt^{2}}\right)}^{2}}dt+\xi \frac{1}{T}\int_{0}^{T}\frac{d\left(F_{m}-F_{d}\right)}{dt}dt$$

where *T* is the sliding window time parameter, and $F_{m}^{x}$, $F_{m}^{y}$, and $F_{m}^{z}$ are the actual interaction forces/moments in the X, Y, and Z directions, calculated by the affected limb and the robot to the end human–computer interaction force $F_{m}$ in the X, Y, and Z directions. $F_{d}$ is the expected human–computer interaction force; that is, when the actual human–computer interaction force and the expected human–computer interaction force remain equal during the observation time, the patient’s motion state is better; when the actual human–computer interaction force between the affected limb and the robot fluctuates more, it means that the patient’s motion state is more unstable, and the *SFR* is larger. The *SFR* value range is limited to (0, 1) by normalization. At the same time, in order to reduce the influence of the weak jitter of the interaction force on the index, a regularization term is added to the *SFR*, and ξ is the regularization coefficient.

Torque efficiency rate (TER), that is,

(12) $$TER=\frac{1}{T}\int_{0}^{T}\left(\frac{F_{m}\cdot F_{d}}{{F_{d}}^{2}}\right)dt$$

where $F_{m}$ is the actual interaction force–torque calculated to the end of the robotic arm, and $F_{d}$ is the expected human–computer interaction force–torque to complete the training task. The closer the actual interaction force is to the expected assist force direction, and the closer the actual interaction force projection is to the expected assist force, indicating that the greater the effective torque output by the affected limb, that is, the better the motion state of the affected limb, the greater the *TER*. The range of *TER* is (0, 1).

The cumulative rate of robot trajectory offset, that is,

(13) $$POR=\frac{\int_{0}^{T}E_{p}dt}{R\cdot T}$$

where $E_{p}$ is the geometric distance of the robot end offset task trajectory, R is the radius of the forced boundary designed in the task, and the robot trajectory cannot exceed the forced boundary. The smaller the cumulative offset of the robot’s end trajectory is, the better the motion state of the affected limb under the current task intensity, and the smaller the *POR*; the *POR* value range is (0, 1).

β is ultimately determined by the above three parameters, that is,

(14) $$\beta =\frac{1}{b_{1}+b_{2}+b_{3}}\cdot \left[b_{1}\cdot POR+b_{2}\cdot SFR+b_{3}\cdot \left(1-TER\right)\right]$$

where *POR* is the robot assistance rate in the *T* time window; *SFR* is the limb state fluctuation rate in the time window; *TER* is the limb torque efficiency in the time window; and $b_{1}$, $b_{2}$, and $b_{3}$ are the weighting factors of the state fluctuation rate and the efficiency, respectively. The better the limb movement state is, the smaller the *SFR,* the greater the *TER*, the smaller the *POR*, and therefore the smaller the β value; that is,β is inversely proportional to the limb state, and the value range is (0, 1).

### 2.3. On-Demand Assist Control Method

**Figure 2:**
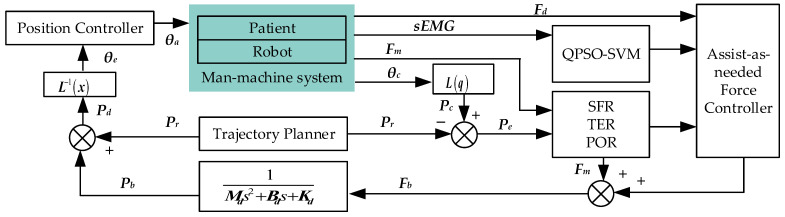
On-demand assist control strategy architecture based on the subjective intent attenuation model.

As shown in Figure 2, the on-demand assist control strategy is based on the admittance model. The goal of the rehabilitation training task is to enable patients to overcome their own limb weight and complete the specified trajectory movement; that is, the expected human–computer interaction force is zero during the training period. The virtual human–computer interaction force is obtained by adding the actual human–computer interaction force and the actual auxiliary force applied by the robot on the affected limb. The virtual human–machine interaction force is converted into positional displacement through the admittance model and is then displayed on the task interface.

Since the real human–computer interaction force contains the active motion intention information of each joint of the patient’s upper limb and the information of its own weight, the expected assist force provided by the robot is to support the affected limb muscle to complete the corresponding rehabilitation training task under the condition that the affected limb muscle does not work. Therefore, the expected output force–torque of the robot is the human–computer interaction force where the limb weight is converted to the end of the robot arm under the current position of the robot arm, that is,

(15) $$\left[\begin{array}{c} F_{d}^{x}\\ F_{d}^{y}\\ F_{d}^{z} \end{array}\right]=L_{3\times 3}(\theta )\cdot C_{3\times 1}$$

Here, $F_{d}^{x}$, $F_{d}^{y}$, and $F_{d}^{z}$ are the expected force–torque in the X, Y, and Z directions, respectively. $L_{3\times 3}(\theta )$ is the joint variable term of the manipulator, and $C_{3\times 1}$ is the characteristic parameter term based on the arm length and body weight of different patients.

If the actual output assist force of the robot is the expected assist force, it can be seen from the control block diagram in Figure 2 above that $P_{d}=P_{e}$. This indicates that under the condition of ignoring the interference of the environment and the friction occurring in the robot joint, the expected assist force is equal to the human–computer interaction force at the end. Even if there is a structured linear relationship between the actual output assist force and the expected assist force, it easily causes patients to rely on the assistive force system. Therefore, the on-demand assist force control parameter regulator is added. Its input is the robot’s expected assist force–torque, the patient’s subjective intention attenuation coefficient α, and the limb training state force position evaluation parameter β, and the output is the robot’s actual output assist force–torque. The formula is as follows:

(16) $$\begin{array}{c} F_{a}^{x}=\left(\alpha -\alpha_{stp}+\beta \alpha_{stp}\right)F_{d}^{x}\\ F_{a}^{y}=\left(\alpha -\alpha_{stp}+\beta \alpha_{stp}\right)F_{d}^{y}\\ F_{a}^{z}=\left(\alpha -\alpha_{stp}+\beta \alpha_{stp}\right)F_{d}^{z} \end{array}$$

where $F_{a}^{x}$, $F_{a}^{y}$, and $F_{a}^{z}$ are the actual assist force–torque of the robot end in the X, Y, and Z directions, respectively; α is the attenuation coefficient of the patient’s subjective intention; $\alpha_{stp}$ is the span of the subjective intention classification; and β is the force position evaluation parameter of the limb training state. The larger the α is, the faster the subjective state of the patient declines, which means that the current task is difficult, and the patient is prone to fatigue. Under the same α coefficient, the smaller the β is, the worse the limb motion state is, and the greater the actual assist force of the robot is. The larger the β is, the better the limb motion state is, and the smaller the actual assist force of the robot is.

## 3. Experimental Research and Analysis

In this study, four healthy volunteers were recruited, aged 25–27 years old, in good physical condition and without basic diseases. There was no significant difference in the general data of the four healthy volunteers (*p* > 0.05). The experiment was conducted at Henan Xiangyu Medical Equipment Co., Ltd (Anyang City, Henan Province, China). All subjects signed informed consent.

### 3.1. Experimental System

**Figure 3:**
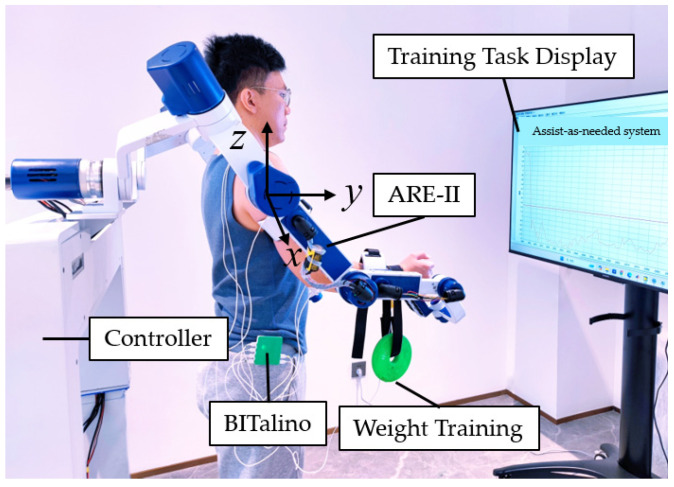
Experimental site.

The experimental interactive system of upper limb rehabilitation on-demand assist control strategy based on the subjective intention attenuation model includes upper limb rehabilitation exoskeleton robot body (ARE-II), sEMG signal acquisition sensor BITalino, control box, virtual scene, and so on. The experimental site is shown in Figure 3. ARE-II has a total of eight degrees of freedom (DOF), which can realize the two-DOF movement of the patient’s scapula, the three-DOF movement of the shoulder joint, the one-DOF movement of the elbow joint, and the two-DOF movement of the wrist joint, and can realize the movement of any posture in the patient’s space. The designed upper limb rehabilitation robot is arranged with a three-dimensional force sensor at the upper and lower arm constraints, respectively, which can directly obtain the interaction force between the upper limb of the patient and the rehabilitation arm.

**Figure 4:**
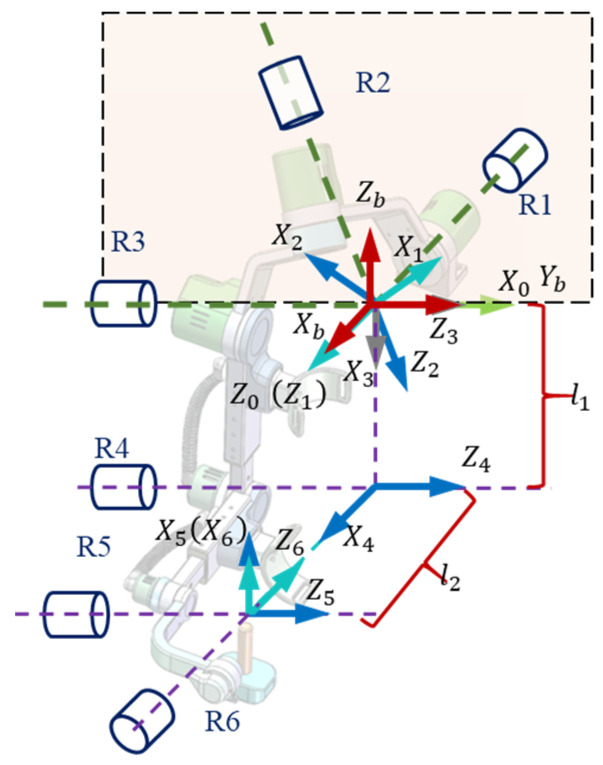
The robot’s right-arm coordinate system.

The upper limb rehabilitation exoskeleton robot (ARE-II) is applied to the rehabilitation training of patients with stroke or spinal cord injury. The left and right are symmetrically arranged. As shown in Figure 4, the length of the mechanical arm is electrically adjustable to meet different sizes of human body. The *Z*-axis of the three joints of the shoulder intersects at one point to form a ball joint. The *Z*-axis of the three adjacent joints of joint 3, joint 4, and joint 5 is parallel. The shoulder of the robot is not three-axis perpendicular to each other. The angle between the three rotation axes of the shoulder joint is set as follows: the angle between joint 1 and joint 2 is 60°, the angle between joint 2 and joint 3 is 60°, and the angle between joint 1 and joint 3 is 90°. A fixed coordinate system $O_{b}-x_{b}y_{b}z_{b}$ is established in the center of the human shoulder joint, and a local fixed coordinate system $O_{0}-x_{0}y_{0}z_{0}$ is established in the center of the shoulder joint of the upper limb rehabilitation robot. In the initial state, the two coordinate systems coincide. The link coordinate system $O_{i}-x_{i}y_{i}z_{i}\;(i=1,2,\ldots ,6)$ is added to each joint of the upper limb rehabilitation robot mechanism, where the $R_{1}$, $R_{2}$, and $R_{3}$ motion axes intersect at the axis of the shoulder joint of the robot arm, and the angle between the $x_{3}$ and $x_{4}$ coordinate axes is 90°. $l_{1}$ and $l_{2}$ correspond to the length of the upper and lower arms of the human upper limb. The three-dimensional force sensor for detecting the human–computer interaction force is located at the *S_1_* point on the big arm-connecting rod and the *S_2_* point on the small arm-connecting rod.

**Figure 5:**
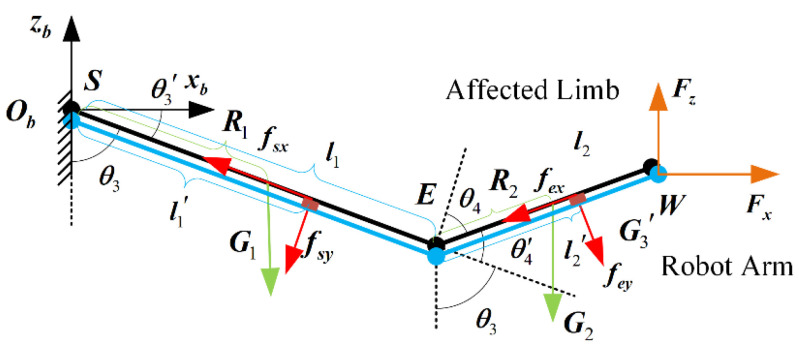
The sagittal in-plane human–computer interaction model.

Since the two three-dimensional force sensors are in direct contact with the patient’s limbs, the collected data are the actual human–computer interaction force. In order to accurately calculate the expected assist force in the training task, it is necessary to separate the information of the patient’s body weight applied to the force sensor. In order to quickly solve the weight of different patients’ limbs, the human–computer interaction model is constrained to move in the sagittal plane, and the human–computer interaction model in the sagittal plane is established as shown in Figure 5. In the figure, $R_{1}$ and $R_{2}$ are the centroid lengths of the affected limbs, respectively. $l_{1}^{\prime }$ and $l_{2}^{\prime }$ are the distance between the sensor and the joint length at the human–computer interaction constraint; $G_{1}$ and $G_{2}$ are the weight of each part of the affected limb; and $f_{sx}$, $f_{sy}$, $f_{ex}$, and $f_{ey}$ are human–computer interaction forces, respectively. $\theta_{3}$ and $\theta_{4}$ are the joint angles of the manipulator, respectively. In order to facilitate the calculation, two angles $\theta_{3}^{\prime }$ and $\theta_{4}^{\prime }$ are defined in the sagittal human–computer interaction model, where $\theta_{3}^{\prime }=-\left(90{}^{\circ}+\theta_{3}\right)$ and $\theta_{4}^{\prime }=90{}^{\circ}-\theta_{4}$.

According to the principle of virtual work, the torque in each joint of the patient’s limb is balanced under the action of its own gravity and the support force of the mechanical arm. Through the analysis of the geometric relationship, the human–computer interaction force $f_{e}$ at the elbow joint is only affected by the weight $G_{2}$ of the patient’s forearm. The torque $M_{e}$ at the elbow joint is obtained as follows:

(17) $$M_{e}=-f_{ey}l_{2}^{\prime }=-G_{2}R_{2}\cos(\theta_{3}^{\prime }+\theta_{4}^{\prime })$$

Similarly, the human–computer interaction force at the shoulder joint is analyzed, and the shoulder joint torque $M_{s}$ is obtained as follows:

(18) $$\begin{array}{l} M_{s}=-f_{ey}(l_{2}^{\prime }+l_{1}\cos\theta_{4}^{\prime })+(-f_{ex}l_{1}\sin\theta_{4}^{\prime })+\left(-f_{sy}l_{1}^{\prime }\right)\\ =-G_{2}R_{2}\cos(\theta_{3}^{\prime }+\theta_{4}^{\prime })-G_{2}l_{1}\cos\theta_{3}^{\prime }-G_{1}R_{1}\cos\theta_{3}^{\prime } \end{array}$$

Then, we have

(19) $$\left[\begin{array}{c} M_{s}\\ M_{e} \end{array}\right]=\left[\begin{array}{cc} -\cos\theta_{3}^{\prime } & -\cos(\theta_{3}^{\prime }+\theta_{4}^{\prime })\\ 0 & -\cos(\theta_{3}^{\prime }+\theta_{4}^{\prime }) \end{array}\right]\left[\begin{array}{c} G_{1}R_{1}+G_{2}l_{1}\\ G_{2}R_{2} \end{array}\right]=L_{2\times 2}(\theta )C_{2\times 1}$$

The joint variable $L_{2\times 2}$ in the above formula is obtained in real time through the joint angles of the robotic arm. The joint torque term $M_{2\times 1}$ is obtained by transforming the human–computer interaction force data with the distance between each sensor and the joint force arm. In order to estimate the limb weight of different users as accurately as possible, the robotic arm drives the affected limb to perform random low-speed motion, during which the affected limb remains relaxed. A total of *k* data are collected in the process, and a more accurate patient characteristic parameter item is obtained as follows:

(20) $${\bar{C}}_{2\times 1}=\frac{1}{k}\sum_{i=1}^{k}L_{2\times 2}{\left(\theta \right)}^{-1}\cdot M_{2\times 1}$$

The relationship between the equivalent interaction force at the end of the manipulator and the joint torque is established as follows:

(21) $$\left[\begin{array}{c} M_{s}\\ M_{e} \end{array}\right]=\left[\begin{array}{cc} -l_{2}\sin\left(\theta_{3}^{\prime }+\theta_{4}^{\prime }\right)-l_{1}\sin\theta_{3}^{\prime } & l_{1}\cos\theta_{3}^{\prime }+l_{2}\cos(\theta_{3}^{\prime }+\theta_{4}^{\prime })\\ -l_{2}\sin\left(\theta_{3}^{\prime }+\theta_{4}^{\prime }\right) & l_{2}\cos(\theta_{3}^{\prime }+\theta_{4}^{\prime }) \end{array}\right]\left[\begin{array}{c} F_{x}\\ F_{z} \end{array}\right]=J^{T}\cdot F$$

In the following, the input parameter of the admittance control strategy of the upper limb rehabilitation robot is the human–computer interaction force–torque signal at the end of the rehabilitation robotic arm. Therefore, the active interaction force of each joint of the rehabilitation robotic arm is transferred to the end of the robotic arm.

(22) $$F={\left(J^{T}\right)}^{-1}M$$

After solving, we have

(23) $$\left[\begin{array}{c} F_{x}\\ F_{y} \end{array}\right]=\frac{\left[\begin{array}{cc} l_{2}\cos(\theta_{3}^{\prime }+\theta_{4}^{\prime }) & -(l_{1}\cos\theta_{3}^{\prime }+l_{2}\cos(\theta_{3}^{\prime }+\theta_{4}^{\prime }))\\ l_{2}\sin(\theta_{3}^{\prime }+\theta_{4}^{\prime }) & -(l_{2}\sin\left(\theta_{3}^{\prime }+\theta_{4}^{\prime }\right)+l_{1}\sin\theta_{3}^{\prime }) \end{array}\right]\left[\begin{array}{c} M_{s}\\ M_{e} \end{array}\right]}{-l_{2}\cos\left(\theta_{3}^{\prime }+\theta_{4}^{\prime }\right)\left(l_{2}\sin\left(\theta_{3}^{\prime }+\theta_{4}^{\prime }\right)+l_{1}\sin\theta_{3}^{\prime }\right)+l_{2}\sin\left(\theta_{3}^{\prime }+\theta_{4}^{\prime }\right)\left(l_{1}\cos\theta_{3}^{\prime }+l_{2}\cos(\theta_{3}^{\prime }+\theta_{4}^{\prime })\right)}$$

The expected assist force of the training task is as follows:

(24) $$F_{d}=J{\left(\theta \right)}^{-1}L_{2\times 2}(\theta )\cdot {\bar{C}}_{2\times 1}$$

**Figure 6:**
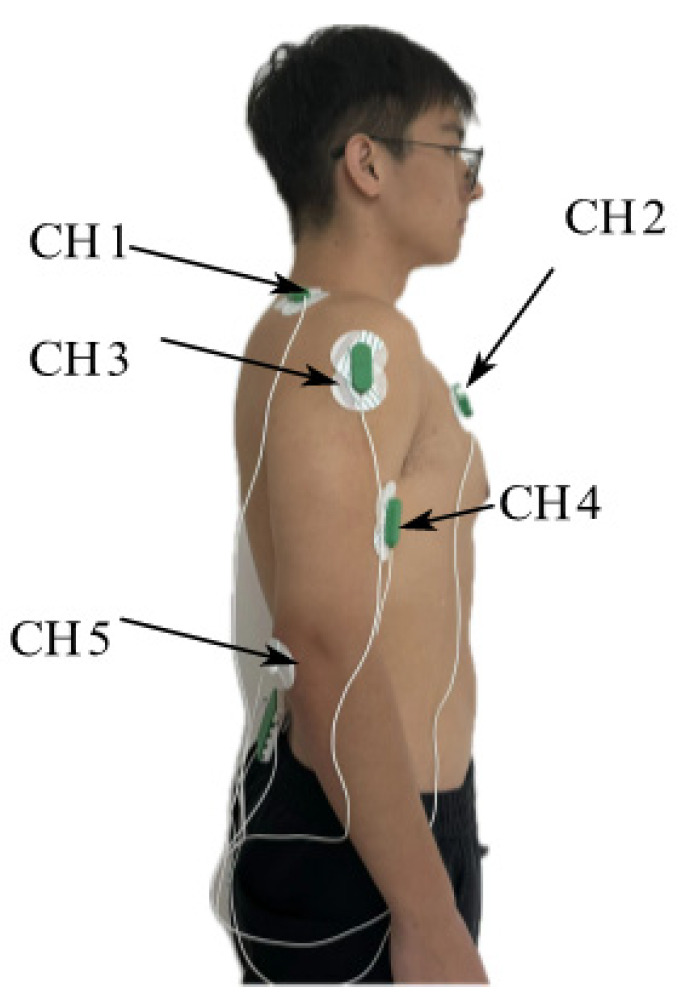
sEMG signal acquisition.

The electromyographic signal acquisition sensor has five channels, and different electrode attachment points are set according to different experimental schemes. As shown in Figure 6, CH1 was set on the upper end of the trapezius muscle, CH2 was set on the pectoralis major muscle, CH3 was set on the deltoid middle bundle, CH4 was set on the biceps brachii, and CH5 was set on the olecranon as the reference electrode. The subjective intention attenuation rate of the subjects was evaluated by measuring the sEMG signal level of the subjects in real time.

The construction of the virtual scene is completed on the Unity3D platform. The main purpose is to increase the fun and immersion of the subjects during training. At the same time, the real position of the robot’s end is displayed on the screen, allowing the subjects to grasp the training situation in real time. The object operated in the designed virtual scene is a mouse, and the patient operates the mouse to walk along an ideal trajectory within a safe area. The coordinates of the mouse and the end of the robotic arm are consistent, setting a task participation scoring mechanism; the closer the value is to the ideal trajectory, the higher the participation score. A 15 min training task was designed, in which participants had to resist the weight of their upper limbs and loads to complete the task. A training score of 60 or above was considered an effective experiment.

### 3.2. Experimental Scheme

In order to verify the effect of this method on the improvement of patients’ initiative, two comparative experiments were set up. In the first experiment, the traditional methods were used, namely, the simple passive training model (M1); the on-demand assist control method (M2) using only the force–position hybrid evaluation index *β*; and the on-demand assist control method (M3) using the method in this paper. Four subjects participated in the experiment. During the experiment, in order to simulate the patient’s state, the subjects loaded a load of 10 pounds on the wrist. The experiment required the subjects’ upper limbs to perform circular trajectory motion in the sagittal plane. In the experiment, the subjects needed to try to “deceive” the robot to obtain higher assist force. The specific experimental scheme is shown in Table 1.

The subjects controlled the upper limb to perform a circular motion trajectory on the sagittal plane. The specific parameters of the experiment were as follows: the center of the circular trajectory (x_0_, z_0_) = (400, −210) and the radius of motion r = 60 mm. At the same time, the admittance control model parameters were set as follows:

(25) $$M_{d}=\left[\begin{array}{c} 0.1\\ 0.1 \end{array}\right],B_{d}=\left[\begin{array}{c} 3\\ 3 \end{array}\right],K_{d}=\left[\begin{array}{c} 225\\ 225 \end{array}\right]$$

At the initial moment of the experiment, the subjective intention attenuation coefficient (α=0.2) and the limb training state force evaluation parameters were set. The moving time window of the subjective intention attenuation was set to 30 s, and the moving speed was 3 s. The moving time window of the training state force evaluation was set to 3 s, and the moving speed was 0.3 s.

During the experiment, the subject’s subjective scoring of the current fatigue state was recorded every 30 s. The fatigue state was divided into three grades. A higher score indicates that the subject experienced more fatigue in the 30 s time window, and the actual assist force of the robot was recorded in real time. In order to reduce the influence of accidental factors on the experimental results, the subjects were required to have enough rest time between the two experiments, and extensive simulation training was carried out before participating in the experiment. The experiment duration was set to 15 min to ensure the subjects entered the fatigue state.

### 3.3. Experimental Results

**Figure 7:**
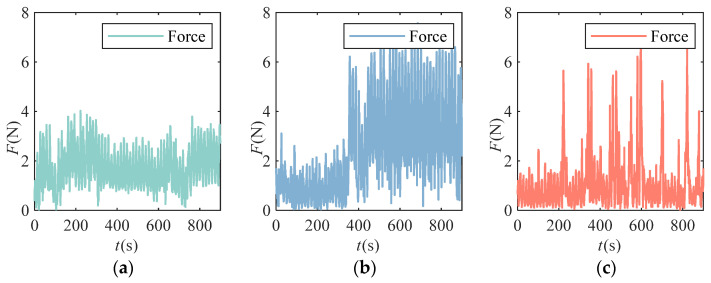
The human–computer interaction force of subject C performing the training task using the three methods: (**a**) eEnd force performance using the M1 traditional control method; (**b**) End force performance using the M2 limb movement assessment method; (**c**) End force performance using the M3 the method of this paper.

When the subjects participated in different comparative experiments, the human–robot interaction force between the subjects and the robot, the joint angle of the manipulator, and the surface sEMG signal of the subjects were continuously collected. In experiment 2, the end force data of subject C completing the three training tasks are shown in Figure 7.

From the diagram, it can be seen that in the M1 training task, the subject completely relaxed the limb, and the mechanical arm drove the limb to complete the corresponding training task. In the M2 training task, the level of human–computer interaction remained low for a period of time at the beginning of the training. At 400 s, the subject was too weak to complete the task. At this time, the human–computer interaction force reached its first peak. At 420 s, the subject was challenged again and worked hard to complete the task goal, so the interaction force decreased rapidly. Then, at 500 s, the subject relied on the assist force provided by the robot and deceived the higher assist force by increasing the end force level and fluctuation frequency. In the M3 training task, the subjects constantly attempted to rely on their own strength to complete the training task, so the human–computer interaction force exhibited multiple peaks. From the original data of human–computer interaction force, it can be seen that only relying on the force position information in training to provide the assist force cannot avoid the patient’s dependence on the robot assist force. Therefore, it is necessary to extract the attenuation trend of patients’ subjective training intention from sEMG signals.

In order to ensure the accuracy of the subjective intention attenuation model and improve the calculation speed of the attenuation coefficient, it was necessary to select three to five features from the existing four-channel sEMG signals as the input of the attenuation model. Therefore, statistical analysis was conducted on these 16 features to obtain the correlation significance and correlation. The average p and F values of the data characteristic quantity compared with each other in the three fatigue states are given in Table 2, where p is the test probability, and F is the influence of random error. When the feature p<0.05 in the table, it means that the feature quantity has a significant difference between different fatigue degrees.

**Figure 8:**
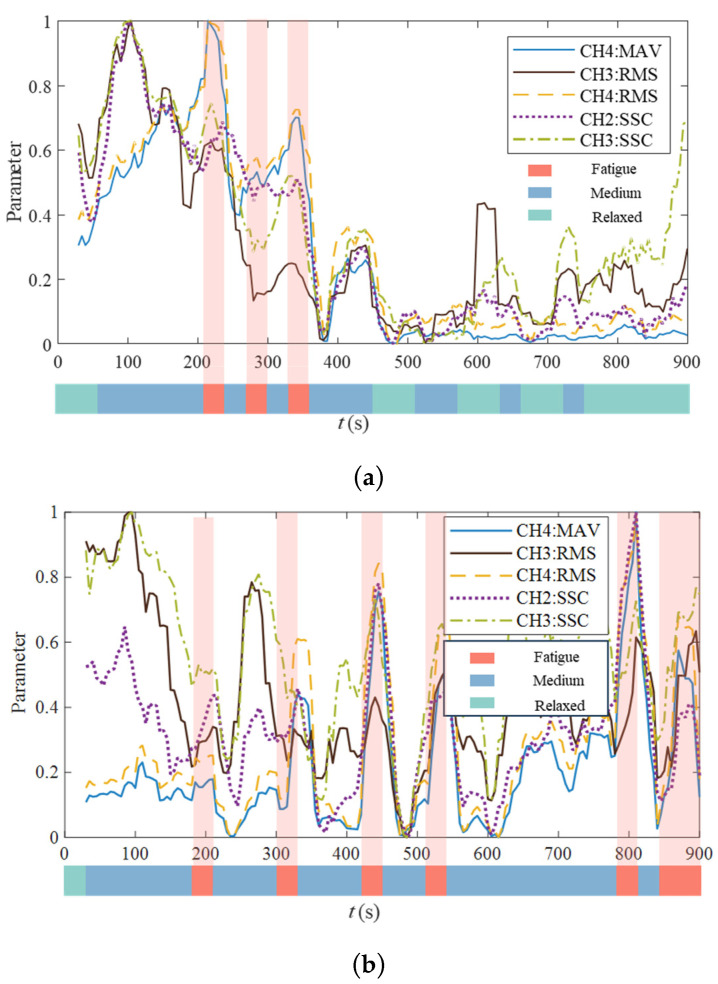
Change curves of sEMG signals’ characteristic quantities in training tasks; (**a**) change curve of the characteristic quantity of subject D performing the M2 training task; (**b**) the change curve of the characteristic quantity of subject D performing the M3 training task.

According to the data listed in the table, CH1 performs poorly. In the comparison of all features in the three fatigue states, F>0.05 in only *MAV* and *SSC*, but the corresponding p<0.05, meaning that although there were significant differences between features, there were still a large number of overlapping areas in the feature data. In the comparison of all the characteristics of CH2 in the three fatigue states, *MAV*, *WL*, and *SSC* features performed better, and their *p* values were all less than 0.05, but in the tired/medium comparison, F>0.05, so only *SSC* was selected as the input to avoid overfitting of the model. In the comparison of all the characteristics of CH3 in the three fatigue states, the *p*-value and *F*-value of *RMS* and SSC were less than 0.05, which were significant and fatigue-related. As an input, the MAV and *RMS* of CH4 had the same distinguishability. Therefore, the *SSC* of CH2, the *RMS* and *SSC* of CH3, and the *MAV* and *RMS* of CH4 were selected as the input of the subjective intention attenuation model. Figure 8a,b show the change curves of the five features extracted by the sEMG signal with time when subject D performs the M2 and M3 training tasks, respectively, intercepting the moment when the subjective intention attenuates the “fatigue” state. During the “fatigue” period, the five feature quantities have a significant improvement trend. At the same time, it can be seen that in the second half of the M2 training task, although the feature quantity also has a trend of improvement, the amplitude is not large, and the corresponding subjective evaluation of the subjects is also in a “medium” or “relaxed” state.

**Figure 9:**
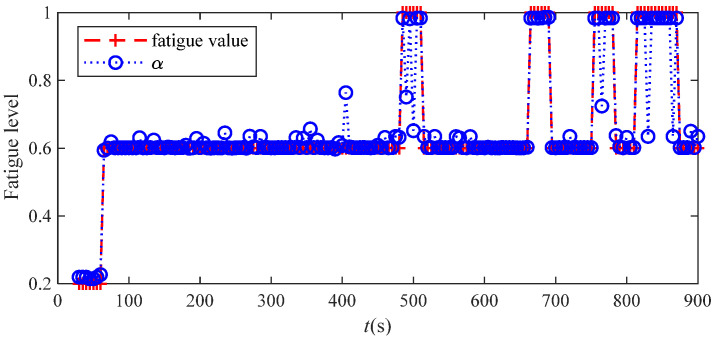
Actual and predicted values of the fatigue state.

In order to verify the effectiveness of the active intention attenuation model, the feature quantities of the five training tasks completed by the four subjects were used as the input parameter $X_{s}$ of the training sample set. At the same time, for subjective fatigue evaluation, data were recorded every 30 s in the training task and were used as the known category information $Y_{s}$ output of the training set, in which the “fatigue” state was set to “1”, the “medium” state was set to “0.6”, and the “easy” state set to “0.2”. The last experimental data were taken as the prediction sample input $X_{l}$ and the real output value *Y* of the prediction sample in the test set. Figure 9 illustrates the actual and predicted values of the fatigue state of the subject $Y_{l}$ participating in the M3 training task. In this experiment, the difference between the predicted value and the true value of 80.57% of the data was within 0.02. If the tolerance range was extended to 0.05, the accuracy of the subjective intention attenuation model could reach 93.14%, and the average accuracy of the subjective intention attenuation recognition of the four subjects reached 87.62%. In order to further analyze the classification effect of QPSO-MLSSVM on volunteer participation status, the minimum MSE, mean MAE, and minimum RMS were used for evaluation, as shown in Table 3.

**Figure 10:**
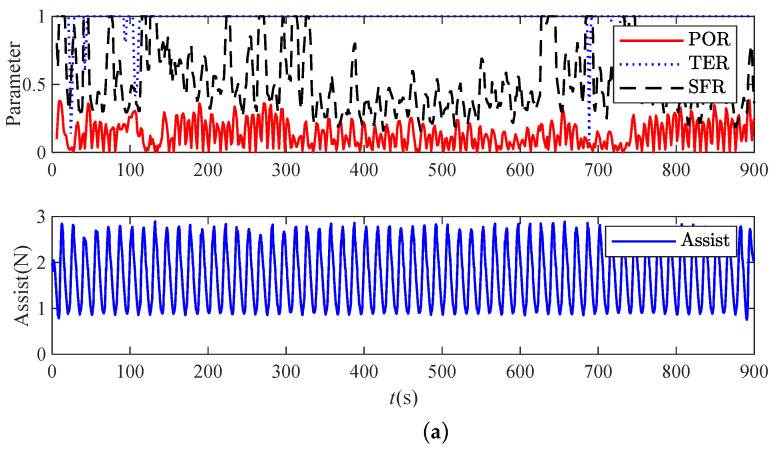

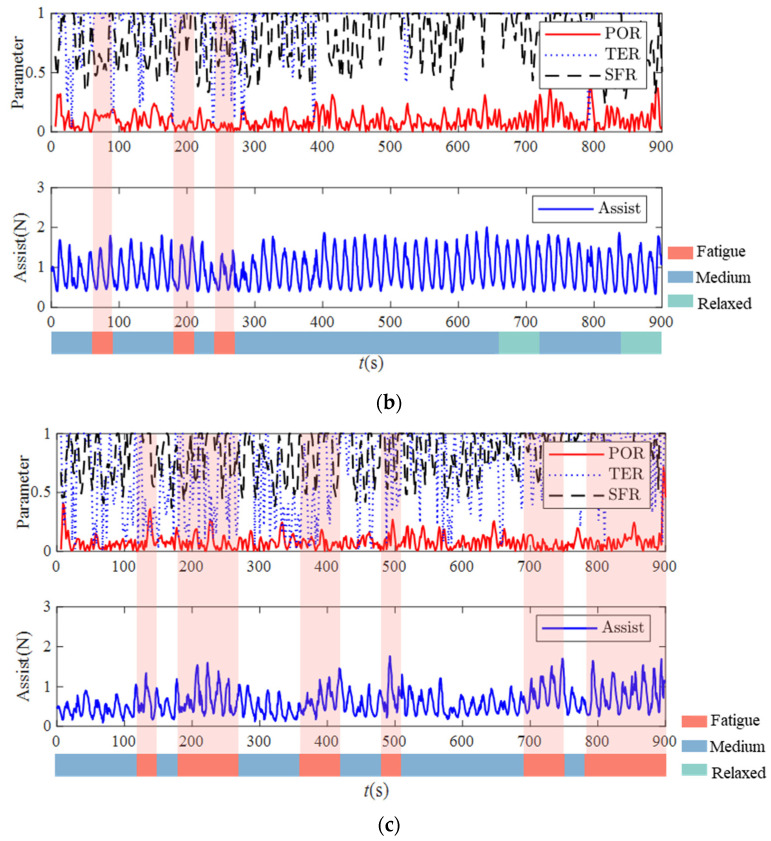
Experimental results under traditional methods: (**a**) experimental results using the traditional method; (**b**) experimental results using the limb movement assessment method; (**c**) the experimental results using the method proposed in this paper.

**Figure 11:**
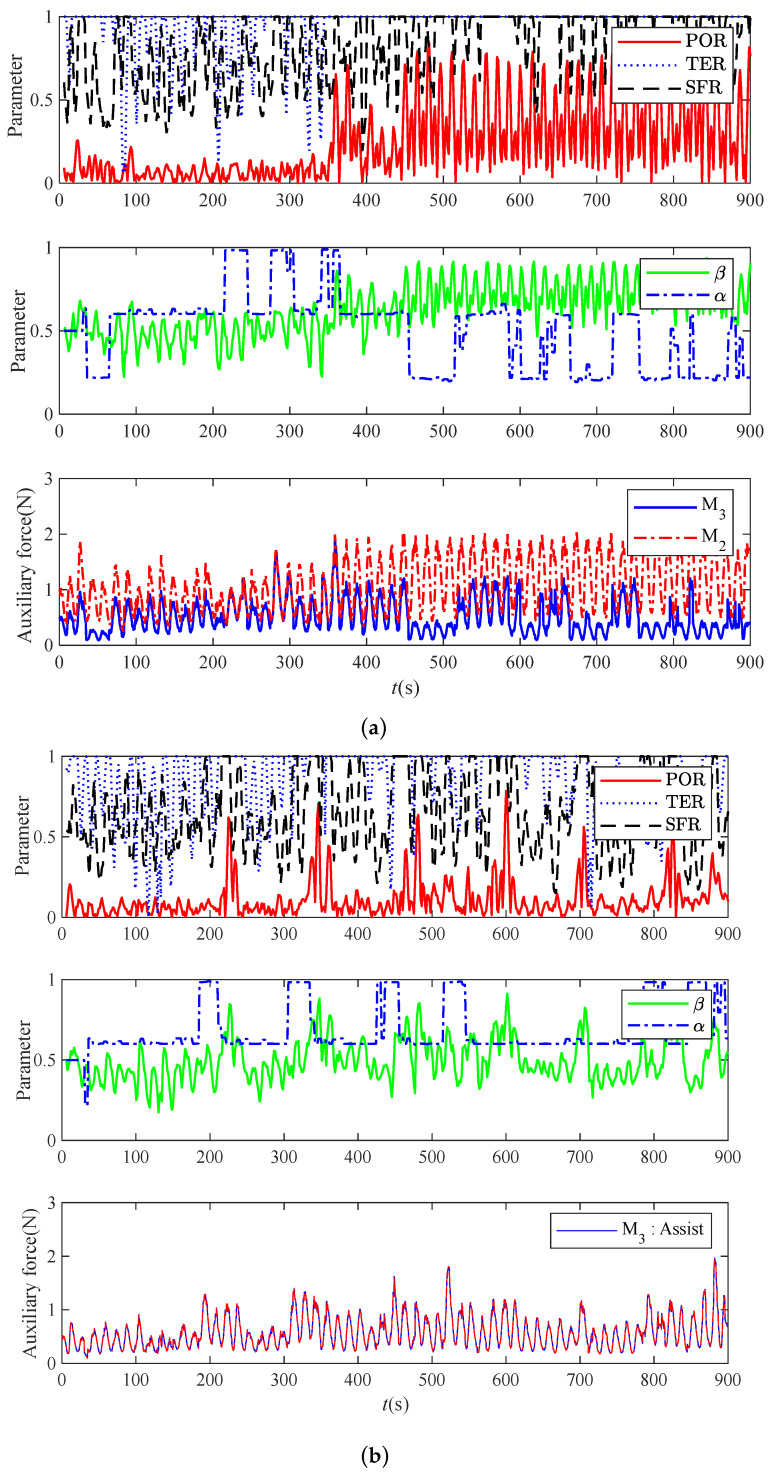
Subject C’s state parameters and robot assist force change curves: (**a**) the experimental results of the M2 training task in experiment 2; (**b**) the experimental results of training tasks using M3.

Considering experiment 1, the results of the active verification experiment of subject A are shown in Figure 10. Regarding experiment 2, the results of the fatigue identification and anti-dependence verification experiment of subject C are shown in Figure 11. Table 4 presents the two experimental results, and the data in the table are the average values of all subjects.

Subject A was selected to analyze the experimental data of three training tasks. In order to eliminate the influence of the assist force dependence phenomenon on the experimental results when subject A performed the M2 experiment, they were required to concentrate as much as possible and rely on their own strength to complete the task when performing the three training tasks.

The analysis chart shows that when the subject’s affected limb was unstable and in the fatigue state, the state parameter values increased; at this time, the average assist force of the M1 training task robot was 1.7335 N; the average assist force of the M2 training task robot was 0.97 N; the average assist force of the M3 training task robot was 0.6116 N; and the corresponding training scores were 98, 80.7, and 83.8, respectively. Compared with the traditional method, the average assist force of the overall training task was reduced by 64.72%. Compared with the assist force method based on force position information, the average assist force was reduced by 36.95%, indicating that the effective active torque of the affected limb greatly increased. At the same time, when the state of the affected limb was unstable, and the subject was in the fatigue state, the average assist force of the robot in this method was 1.0341 N, and the average assist force of the robot using M2 was 0.9614 N. Compared with the assist force, the two decreased slightly, but the decrease was not large, indicating that the method in this paper can provide sufficient assist force in the fatigue state.

In experiment 2, which was performed in order to verify the phenomenon of assist force dependence in the M2 training task, subject C had to try to “deceive” the robot to generate more assist force when performing this task. At the same time, in order to verify that the subjective intention attenuation model of M3 can prevent the phenomenon of assist force dependence, the M3 method was used to simulate and compare the data in the M2 training task. Figure 11 shows the performance of subject C in trying to “deceive” the robot to provide more assistive force when performing the M3 training task.

The analysis of the chart shows that when subject C performed the M2 training task. Starting from 450 s, in order to “deceive” the robot to increase the auxiliary force, a deliberately intermittent force was generated to create the phenomenon of unstable motion. The robot increased the average auxiliary force to 1.2313 N. Using M3 to reanalyze the data, the average assist force of the corresponding robot did not increase but rather decreased to 0.6242 N, corresponding to the actual fatigue state of the subject. Therefore, the average assist force was reduced by 49.31% compared with the average assist force of M2. As shown in Figure 11b, although subject C tried to “deceive” the robot several times, the assistive force did not improve.

Considering the results of subjects C/D participating in the M2 and M3 experiments, the average scores of the tasks and the proportion of subjective fatigue state of the subjects throughout the entire training period are shown in Table 5. In order to compare the improvement effect of the on-demand assistive force control strategy on patient initiatives, subjects C/D also underwent five training tasks without the assistive admittance control strategy. By comparison, it can be seen that the average scores of M2 and M3 training tasks were 86.3 and 85.5, respectively, with little difference. However, the proportion of fatigue in M3 was much higher than that in M2. It can be seen that under the same task score, the subjective participation of patients in the M3 task was greater. Comparing the M1 task without assistance and the M3 task, it can be seen that the fatigue rate of M1 without assistance was slightly higher than that of M3, but the average score of M1 without assistance was much lower than that of M3. This is mainly because the subjects had to have more initiative to complete the M1 task without assistance training, and their physical strength decreased faster, which could not support the completion of the training task.

## 4. Discussion

The assist-as-needed rehabilitation training control strategy based on the subjective intention attenuation model in this study aimed to enhance the subjective intention attenuation model and the assist force control model based on the force position information on the traditional admittance model. According to the motion state of the affected limb and the real fatigue state of the patient, the assist force of the robot in the affected limb was adaptively adjusted to improve the initiative of the patient to participate in the training. Through the analysis of two groups of comparative experiments, it can be seen that the average assist force provided by this method was 67% lower than that provided by the traditional method, and the average assist force provided by the on-demand assist training strategy based on the assist force control model using only the force position information was reduced by 16%, thus improving the initiative of the affected limb. We used the method described in this article to avoid situations where patients have not entered the fatigue stage, but the system provides excessive assistance. This method can identify the fatigue state of patients to prevent the occurrence of dependency phenomenon. In experiment 2, the fatigue state recognition rate of M3 reached 93.41%, and the average auxiliary force decreased by 48.8% compared to M2. Therefore, the method in this paper can effectively improve the initiative of the affected limb and meet the requirements of rehabilitation training for the upper limb rehabilitation training system.

Finally, in the study of the method in this paper, two interesting phenomena were found. First, as shown in Figure 8, at the beginning of training, the sEMG signals revealed often relatively high values. If the judgment is based on the subjective intention attenuation model, this indicates a fatigue state. However, due to physical reserve and other reasons, the subjects were not involved in fatigue evaluation in real tasks, so how to determine the emergence of the first fatigue state is the next problem to be solved. Secondly, in the process of processing training data, it was found that the subjects needed a higher assist force and there was a correlation between the trajectory position and speed. Due to space limitations, this work was not carried out here but is planned as a follow-up study.

## Figures and Tables

**Table 1:** Experimental scheme.

Subject	Method	Condition	Purpose
A/B	M1: Traditional methods	Complete 15 min trajectory training	Compare the control performance of three methods
	M2: Limb movement assessment method		
	M3: The method of this paper		
C/D	M2: Limb movement assessment method	Try to “deceive” the robot to provide more assistance	Compare two methods to reduce patient dependence
	M3: The method of this paper		

**Table 2:** Comparison of the significance of characteristic quantities.

Channel Number	Difficulty Comparison	MAV		RMS		WL		SSC	
		*p*	*F*	*p*	*F*	*p*	*F*	*p*	*F*
CH1	Tired/ Medium	0.001	0.1592	0.0962	0.1587	0.0001	0.2766	3.7 × 10^−9^	0.4826
	Tired/Easy	3.24 × 10^−7^	0.2281	2.27 × 10^−8^	0.2099	2.68 × 10^−7^	0.2832	2.17 × 10^−7^	0.7296
	Medium/Easy	2.45 × 10^−11^	0.0002	0.0962	0.1587	0.0001	0.2766	3.78 × 10^−9^	0.4826
CH2	Tired/ Medium	0.0134	0.7790	0.2393	0.6471	0.0145	0.8479	0.0033	0.2153
	Tired/Easy	7.59 × 10^−13^	0.0214	3.21 × 10^−15^	0.9351	1.95 × 10^−13^	0.0001	1.26 × 10^−18^	0.0041
	Medium/Easy	2.57 × 10^−17^	0.0051	2.99 × 10^−7^	0.4950	1.34 × 10^−11^	2.5 × 10^−6^	6.03 × 10^−15^	2.5 × 10^−7^
CH3	Tired/ Medium	9.26 × 10^−6^	6.2 × 10^−6^	0.0002	2.12 × 10^−5^	5.23 × 10^−6^	6.83 × 10^−7^	0.0011	1.1 × 10^−7^
	Tired/Easy	1.08 × 10^−9^	0.9632	1.2 × 10^−6^	0.0511	1.16 × 10^−8^	0.5378	1.49 × 10^−18^	0.0008
	Medium/Easy	7.46 × 10^−12^	1.8 × 10^−8^	7.13 × 10^−10^	0.0043	1.35 × 10^−11^	2.98 × 10^−8^	1.64 × 10^−8^	0.0158
CH4	Tired/ Medium	0.0497	7.38 × 10^−5^	5.44 × 10^−5^	0.0058	0.0749	0.0031	0.031274	0.0001
	Tired/Easy	1.8 × 10^−21^	3.02 × 10^−22^	4.07 × 10^−24^	3.59 × 10^−18^	1.07 × 10^−22^	1.43 × 10^−15^	1.4 × 10^−20^	0.0046
	Medium/Easy	2.16 × 10^−16^	1.94 × 10^−16^	1.7 × 10^−18^	1.13 × 10^−15^	1.33 × 10^−16^	1.76 × 10^−12^	1.92 × 10^−14^	0.3813

**Table 3:** The error between the predicted and real values of the model.

Title 1	MSE	MAE	RMS
Matching error	1.2104 × 10^−4^	0.0191	0.0582

**Table 4:** The experimental results of limb initiative verification.

Subject	Method	Average Assist (N)	Reduction Rate of Assist (%)	Fatigue Recognition Rate (%)
A/B	M1: Traditional methods	1.745	48.8%	93.41%
	M2: Limb movement assessment method	0.9247		
	M3: The method of this paper	0.5992		
C/D	M2: Limb movement assessment method.	1.0641		
	M3: The method of this paper	0.5794		

**Table 5:** The scores of on-demand limb training tasks and proportion of subjective fatigue status.

Subject	Method	Average Score	Proportion of Fatigue State (%)
C/D	M2: Limb movement assessment method	86.3	6
	M3: The method of this paper	85.5	30.67
	M1 without assistance: Traditional methods	48	44.78

## Data Availability

The original contributions presented in the study are included in the article, further inquiries can be directed to the corresponding author/s.
